# Predisposing and Precipitating Risk Factors for Delirium in Elderly Patients Admitted to a Cardiology Ward: An Observational Cohort Study in 1,042 Patients

**DOI:** 10.3389/fcvm.2021.686665

**Published:** 2021-09-29

**Authors:** Carl Moritz Zipser, Florian Freimut Hildenbrand, Bernhard Haubner, Jeremy Deuel, Jutta Ernst, Heidi Petry, Maria Schubert, Katja-Daniela Jordan, Roland von Känel, Soenke Boettger

**Affiliations:** ^1^Consultation-Liaison Psychiatry and Psychosomatic Medicine, University of Zurich, University Hospital Zurich, Zurich, Switzerland; ^2^Department of Neurology and Neurophysiology, University of Zurich, Balgrist University Hospital, Zurich, Switzerland; ^3^Department of Gastroenterology, University of Zurich, University Hospital Zurich, Zurich, Switzerland; ^4^Department of Cardiology, University of Zurich, University Hospital Zurich, Zurich, Switzerland; ^5^Stem Cell Institute, University of Cambridge, Cambridge, United Kingdom; ^6^Department of Hematology, University of Zurich, University Hospital Zurich, Zurich, Switzerland; ^7^Center for Clinical Nursing Science, University of Zurich, University Hospital Zurich, Zurich, Switzerland; ^8^Zurich University of Applied Science, School of Health Professions, Winterthur, Switzerland

**Keywords:** delirium, cardiology, predisposing factors, precipitating factors, personalized medicine

## Abstract

**Aim:** Although the risk factors for delirium in general medicine are well-established, their significance in cardiac diseases remains to be determined. Therefore, we evaluated the predisposing and precipitating risk factors in patients hospitalized with acute and chronic heart disease.

**Methods and Results:** In this observational cohort study, 1,042 elderly patients (≥65 years) admitted to cardiology wards, 167 with and 875 without delirium, were included. The relevant sociodemographic and cardiac- and medical-related clusters were assessed by simple and multiple regression analyses and prediction models evaluating their association with delirium. The prevalence of delirium was 16.0%. The delirious patients were older (mean 80 vs. 76 years; *p* < 0.001) and more often institutionalized prior to admission (3.6 vs. 1.4%, *p* = 0.05), hospitalized twice as long (12 ± 10 days vs. 7 ± 7 days; *p* < 0.001), and discharged more often to nursing homes (4.8 vs. 0.6%, *p* < 0.001) or deceased (OR, 2.99; 95% CI, 1.53–5.85; *p* = 0.003). The most relevant risk factor was dementia (OR, 18.11; 95% CI, 5.77–56.83; *p* < 0.001), followed by history of stroke (OR, 6.61; 95% CI 1.35–32.44; *p* = 0.020), and pressure ulcers (OR, 3.62; 95% CI, 1.06–12.35; *p* = 0.040). The predicted probability for developing delirium was highest in patients with reduced mobility and institutionalization prior to admission (PP = 31.2%, *p* = 0.001). Of the cardiac diseases, only valvular heart disease (OR, 1.57; 95% CI, 1.01–2.44; *p* = 0.044) significantly predicted delirium. The patients undergoing cardiac interventions did not have higher rates of delirium (OR, 1.39; 95% CI 0.91–2.12; *p* = 0.124).

**Conclusion:** In patients admitted to a cardiology ward, age-related functional and cognitive impairment, history of stroke, and pressure ulcers were the most relevant risk factors for delirium. With regards to specific cardiological factors, only valvular heart disease was associated with risk for delirium. Knowing these factors can help cardiologists to facilitate the early detection and management of delirium.

## Introduction

Delirium is the most prevalent acute neuropsychiatric syndrome in elder general hospital populations, with a relevant short- and long-term impact on individual health and health care costs (1–3). It is characterized by an abrupt onset and fluctuating course of disturbances in attention, awareness, and disturbance in cognition due to an underlying precipitant or, more commonly, several precipitants (4, 5). The pathophysiology of delirium is hypothesized to result from cortical network dysfunction and neurotransmitter imbalance (6). In the general hospital setting, delirium rates range from 10 to 60%, and in medical departments from 18 to 35% (7, 8). In cardiology, the overall prevalence ranges from 15% to more than 50% in patients 85 years or older (9). In cardiac patients, delirium is associated with prolonged hospitalization, poorer functional outcomes, and higher in-hospital and 6-month mortality as well as a higher need for institutionalization and longer hospital stay (10–14). In patients admitted to internal medicine services, advanced age, frailty, and cognitive impairment are the most relevant predisposing risk factors, while infection, electrolyte disturbance, and acute renal failure may precipitate delirium among many other factors (3, 15). Both predisposing and precipitating factors interact: in patients with low predisposition, the precipitating factors have to be more severe than in patients with high predisposition, in which less severe noxious insults cause delirium (16). Although the interrelation remains incompletely understood, epidemiology indicates that delirium may accelerate cognitive decline and dementia and that patients with dementia likely develop delirium (17, 18).

Although the predisposing and precipitating factors for delirium in patients admitted to general internal medicine services are known, their role in patients with cardiac diseases remains to be elucidated. Therefore, the aim of this study was to investigate the predisposing and precipitating risk factors in patients admitted to a cardiology ward. For this purpose, these factors were analyzed in an observational cohort of 1,042 elderly patients admitted to a cardiology ward in the acute hospital setting of a tertiary university hospital.

## Methods

### Patients, Measurements, and Procedures

**Figure 1:**
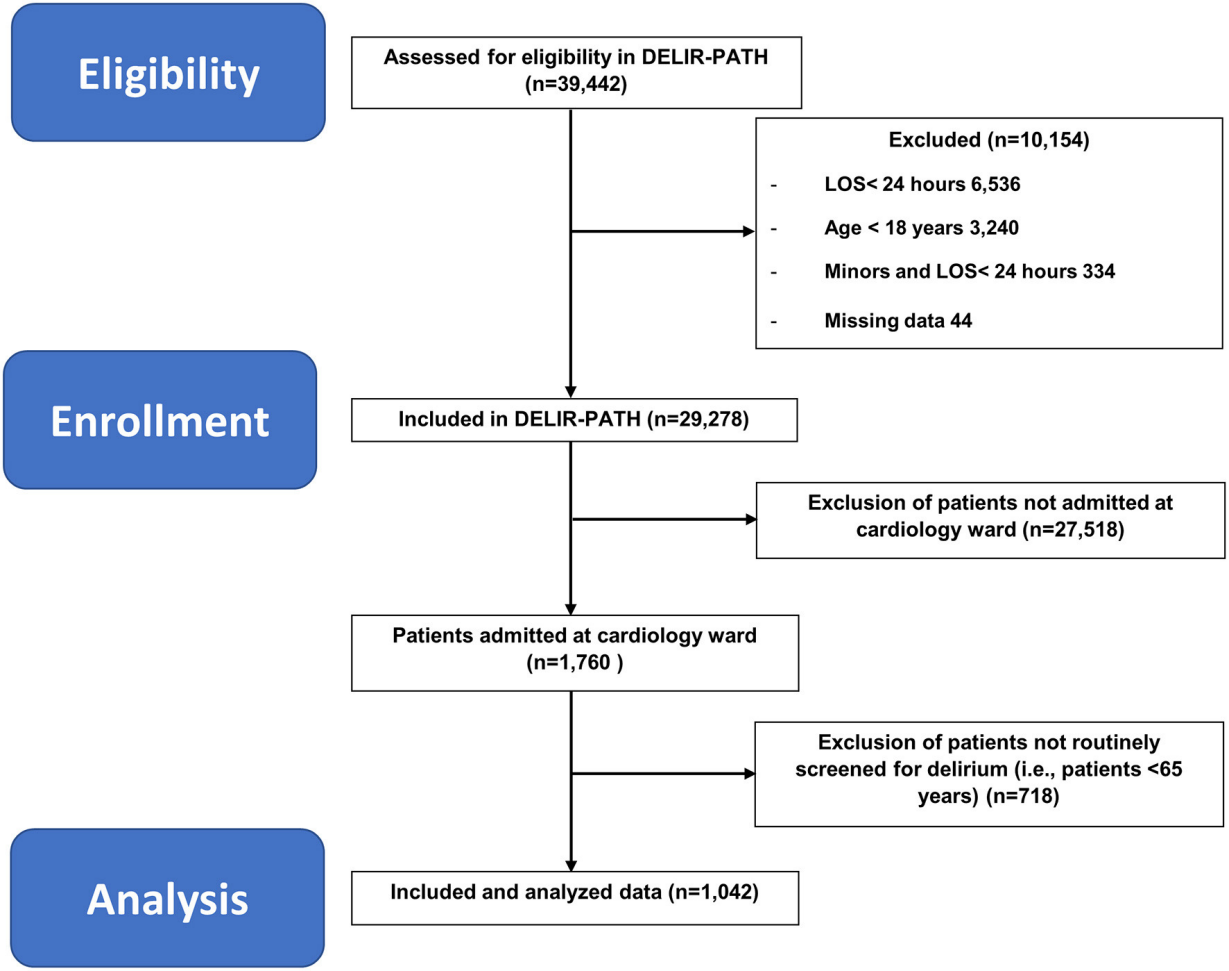
STROBE flow chart of sample recruitment. DELIR-PATH, Detect Evaluate Control Inpatient Risk factors, Prevent And Treat Hospital-Acquired Deliriums; LOS, length of stay.

All patients in this observational cohort study were retrieved from the DELIR-PATH (Detect Evaluate Control Inpatient Risk factors, Prevent And Treat Hospital Acquired Deliriums), a health service research and practice development project at the University Hospital Zurich aimed to improve the prevention, early detection, and management of delirium in all hospitalized patients (19). The cohort in this study refers to delirium screening data collected between January 1 to December 31, 2014. In total, 39,442 patients were enrolled in the Delir-Path Health Service Research Project. The exclusion criteria were age <18 years, length of stay <24 h as well as missing data, resulting in 29,278 eligible patients. Of these, 1,042 elderly patients (≥65 years) admitted to the cardiology ward and routinely screened for delirium were included in this study (see Figure 1 for the STROBE flow chart of sample recruitment). Patients under 65 years were not included in this analysis because they were not routinely screened.

The Delir-Path implements a screening algorithm with the Delirium Observation Screening Scale (DOS) (20). The DOS is a 13-item scale validated to detect the presence and severity of delirium in accordance with the DSM-IV criteria (21).

Screening and scoring were performed by trained nursing staff (all bedside nurses were trained). The training included 4-h courses with mandatory prior eLearning and literature research, with a subsequent test of learning success (multiple choice test). The participants were educated with case reports, state-of-the-art lectures on delirium epidemiology and pathophysiology as well as the diagnostic criteria of delirium and trained in obtaining delirium scores (theoretical procedure of scoring, practical lessons with video samples, and role playing). Additionally, the departments were supported by the delirium task force during the initiation period of the study and continuously on-demand throughout the study. The DOS was administered three times per day during the first 3 days of admission for all patients in this cohort. The cutoff of ≥3 is commonly used (22), as it has good screening performance, with a sensitivity of 0.89, a specificity of 0.88, and a high negative predictive value for delirium of almost 100% (23). The patient data presented here include patients from the cardiology ward. Cardiological interventions include catheter interventions, but not cardiac surgery, because those patients are routinely managed on cardiac ICU.

### Classification of Diagnostic Clusters

Relevant data on medical diagnoses for this sub-study was automatically retrieved from the electronic medical chart (Klinikinformationssystem, KISIM, CisTec AG, Zurich). For the purpose of this analysis, diagnostic clusters according to the 10th revision of the International Classification of Diseases (ICD-10) (24), displayed in Supplementary Table 1, were created. For this purpose, equivalent clusters from different chapters were pooled, e.g., dementia from the psychiatric chapter F00-03 and the respective neurological chapter—G30-32, other degenerative diseases of the central nervous system (CNS), or ischemic insults/strokes—G46 and I63, for instance, the cluster of substance use disorders (ICD-10 F10-19) comprised acute and chronic disorders associated with the use of alcohol, opioids, cannabinoids, and other drugs. The cluster of atherosclerosis (I70) included aortic, renal, and limb atherosclerosis but excluded coronary, mesenterial, pulmonal, and cerebral atherosclerosis. In total, 18 general medical and specific cardiac clusters were created; four factors were related to admission and hospitalization and three to functional impairment (see Table 1). Reduced mobility was detected with the electronic Nursing Assessment—Acute Care motion item “activity” and was dichotomized in regular mobility vs. either reduced, assisted, or no mobility.

**Table 1:** Simple regression analysis of factors associated with delirium.

	**Delir *n* (%)**	**Non-Delir *n* (%)**	***P***
*Total n*	167	875	
***Admission/hospitalization***			
- Institutionalized prior to admission	95 (56.9%)	447 (51.1%)	0.177
- Emergency admission	43 (25.7%)	109 (12.5%)	<0.001^*^
- Cardiac intervention	65 (38.9%)	212 (24.2%)	<0.001^*^
- Intensive care management	42 (25.1%)	75 (8.6%)	<0.001^*^
***Functional impairment***			
- Reduced mobility	155 (92.8%)	594 (67.9%)	<0.001^*^
- Hearing impairment	71 (42.5%)	190 (21.7%)	<0.001^*^
- Visual impairment	100 (59.9%)	231 (26.4%)	<0.001^*^
***Psychiatric disorders***			
- Dementias/degenerative disorders	13 (7.8%)	6 (0.7%)	<0.001^*^
- Substance use disorder	15 (9.0%)	26 (3.0%)	0.001^*^
***Cardiovascular diseases***			
- Cardiomyopathy	14 (8.4%)	63 (7.2%)	0.628
- Cardiac arrest	6 (3.6%)	14 (1.6%)	0.115
- Heart failure	84 (50.3%)	301 (34.4%)	<0.001^*^
- Valvular heart disease	74 (44.3%)	234 (26.7%)	<0.001^*^
- Ischemic heart disease	113 (67.7%)	591 (68.2%)	1.0
- Atherosclerosis	24 (14.4%)	93 (10.6%)	0.180
- Cerebrovascular insult	5 (3.0%)	3 (0.3%)	0.004^*^
***Respiratory diseases***			
- Respiratory disease	19 (11.4%)	67 (7.7%)	0.124
- Pleural effusions	8 (4.8%)	11 (1.3%)	0.006^*^
***Infectious disease***			
- Sepsis/SIRS	12 (7.2%)	20 (2.3%)	0.002^*^
- Pneumonia	4 (2.4%)	7 (0.8%)	0.084
- Cystitis	32 (19.2%)	115 (13.1%)	0.051
***Others***			
- Liver failure	4 (2.4%)	5 (0.6%)	0.042^*^
- Male sex	90 (53.9%)	555 (63.4%)	0.024^*^
- Age ≥80 years	87 (52.1%)	262 (29.9%)	<0.001^*^
- Kidney disease	68 (40.7%)	324 (37.0%)	0.384
- Electrolyte disturbances	7 (4.2%)	65 (7.4%)	0.138
- Pressure ulcers	10 (6.0%)	6 (0.7%)	<0.001^*^

*The values are given as absolute values (n=) and percentages of total n*.

* *A p <0.05 is considered statistically significant. SIRS, Systemic inflammatory response syndrome*.

### Statistical Methods

Data were analyzed with the Statistical Package for the Social Sciences, version 25, for Windows. The characteristics of the sample were summarized using means and standard deviations (when the distribution was normal) or medians and interquartile ranges (when the distribution was not normal) for continuous variables or ordinal variables and percentages for categorical variables. The data set was dichotomized according to the presence or absence of delirium. Further dichotomizations were made on the number of interventions ≥1 vs. 0 (i.e., catheter interventions) and residence status prior to admission—institution vs. home. The normality of the data distribution was tested with the Shapiro–Wilk test. Between-group differences for continuous variables were computed using Student's *t*-test (when the distribution was normal) and Mann–Whitney *U*-test (when the distribution was not normal) and for categorical variables with Pearson's χ^2^ (when the test sample, *n*, was >20) or Fisher's exact test (when the test sample was ≤ 20).

For the risk factor analysis, in a first step, sociodemographic and medical characteristics were analyzed with simple logistic regressions stratified by delirium presence and absence. Then, for the evaluation of the relevance of individual diagnostic clusters, multiple logistic regressions including ORs and CIs were calculated, with the dependent variable set on the presence or absence of delirium and the respective diagnoses clusters treated as covariates. The model was optimized with Cox–Snell's and Nagelkerke's *r*^2^. All variables collected are shown in Table 1. To account for symptom constellations associated with delirium, we computed decision trees. The decision trees were created with CHAID growing method, and the presence of delirium was the dependent variable, with predisposing and precipitating factors that were significant in the multivariate analysis as independent variables.

## Results

### Baseline Characteristics of the Cardiac Patients

The prevalence of delirium present at admission or developing within the first 3 days of hospitalization in patients admitted to the cardiology ward was 16.0% (167/1,042). The delirious patients were older (80 ± 7 vs. 76 ± 7 years old, *P* < 0.001) and more often male (53.9%; *P* = 0.024). The delirious patients were less commonly admitted from home (71 vs. 86%, *P* = 0.001) but were more likely to be transferred from referring hospitals for specialized treatment (OR, 2.0; 95% CI, 1.32–3.03; *P* = 0.002) or from nursing homes (OR, 2.68; 95% CI, 0.99–7.24; *P* = 0.05). In total, the hospital stay was twice as long in patients with delirium than in those without (12 ± 10 vs. 7 ± 7 days). At discharge, the delirious patients returned to home less often than the non-delirious patients (36 vs. 77%, *P* < 0.001). The delirious patients were often discharged to nursing homes (OR, 8.75; 95% CI, 2.83–27.10; *P* < 0.001), other hospitals (OR, 3.21; 95% CI, 2.11–4.86; *P* < 0.001), or rehabilitation (OR, 3.12; 95% CI, 2.06–4.73; *P* < 0.001). The patients with delirium died more often during hospitalization (OR, 2.99; 95% CI, 1.53–5.85; *P* = 0.003). Detailed demographics are presented in Table 2.

**Table 2:** Sociodemographic and medical characteristics of cardiac patients with and without delirium.

	**Delirium *n* (%)**	**No delirium *n* (%)**	**P, OR, CI95%**
Total *n*	167	875	
Age (in years)^*^	80.0, 7.0	76.3, 7.1	<0.001^a^
***Gender (in %)***			
Male	90 (53.9%)	551 (63.4%)	0.024^b^
Female	77 (46.1%)	324 (36.6%)	0.024^b^
***Admitted from (in %)***			
Home	119 (71.3%)	752 (85.9%)	0.001, 0.41, 0.28–0.60^b^
Hospital	37 (22.2%)	105 (12.5%)	0.002, 2.0, 1.32–3.03^b^
Nursing home	7 (3.6%)	9 (1.4%)	0.05, 2.68, 0.99–7.24^b^
Other	4 (3.0%)	9 (0.9%)	0.043, 3.34, 1.08–10.35^b^
***Mode of admission (in %)***			
Emergency	95 (56.9%)	446 (51.1%)	0.177, 1.26, 0.90–1.76^b^
Elective	58 (34.7%)	385 (43.9%)	0.033, 0.68, 0.48–0.96^b^
Length of stay (in days)^*^	12.5, 10.4	6.9, 6.9	<0.001^a^
Intensive care management	42 (25.1%)	79 (8.6%)	<0.001, 3.58, 2.35–5.47^b^
>24 h ventilated	18 (10.8%)	35 (4.0%)	0.001, 2.90, 1.60–5.25^b^
> one intervention performed	65 (38.9%)	210 (24.2%)	<0.001, 1.99, 1.41–2.82^b^
***Discharged to (in %)***			
Home	60 (35.9%)	674 (76.9%)	<0.001, 0.17, 0.12–0.24^b^
Nursing home	8 (4.8%)	9 (0.6%)	<0.001, 8.75, 2.83–27.10^b^
Other hospital	42 (25.1%)	87 (9.5%)	<0.001, 3.21, 2.11–4.86^b^
Rehabilitation	42 (25.1%)	87 (9.7%)	<0.001, 3.12, 2.06–4.73^b^
Deceased	13 (8.4%)	26 (3.0%)	0.003, 2.99, 1.53–5.85^b^

* *Mean, standard deviation*,

a *Mann Whitney U-test*,

b *Pearson's or Fisher's. p <0.05*.

### Predisposing and Precipitating Factors for Delirium in Cardiac Patients

**Figure 2:**
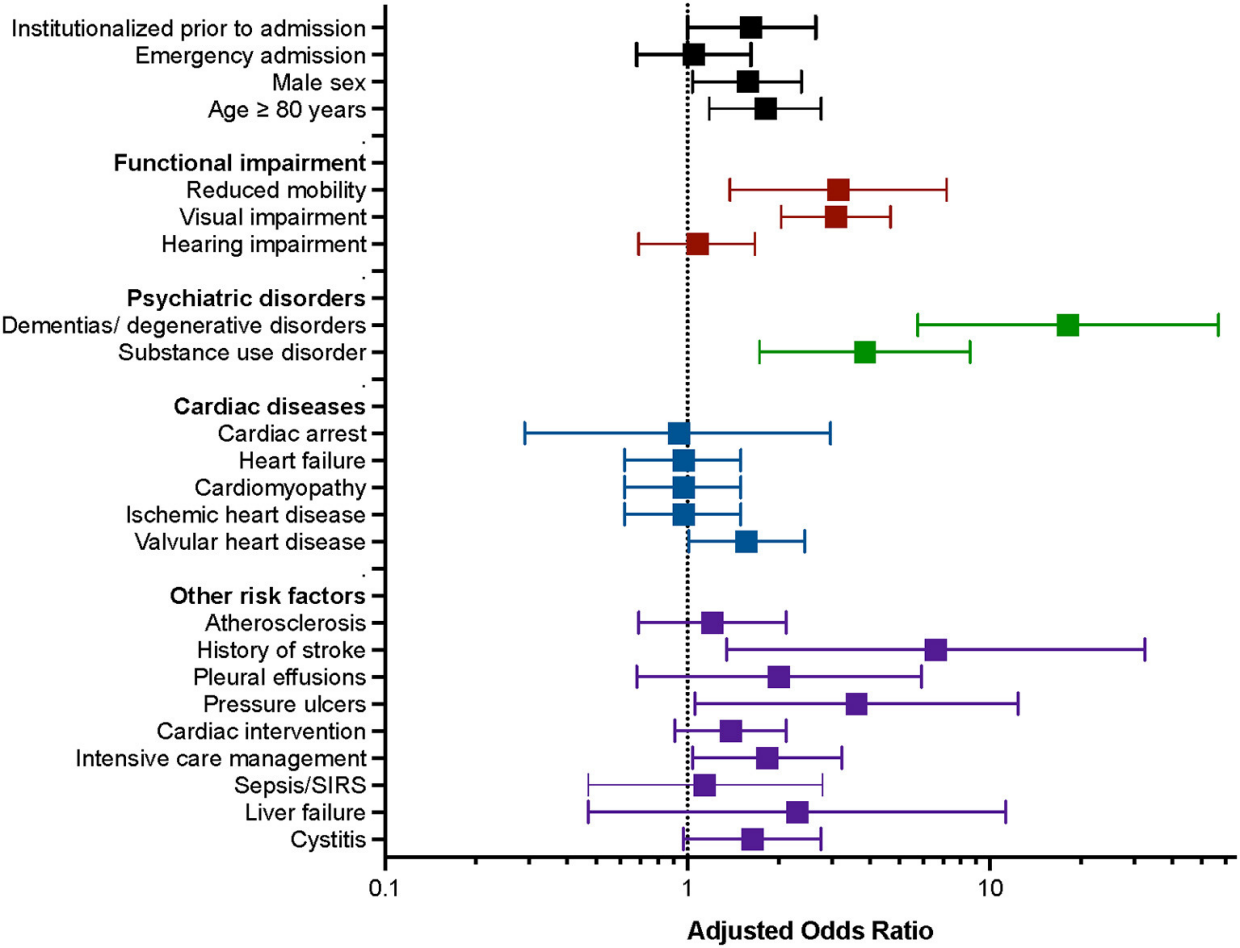
Graphic representation of the multiple regression model of predisposing and precipitating factors for the development of delirium in patients admitted at the cardiology ward. Predisposing and precipitating factors are grouped by demographic factors, functional impairment, psychiatric disorders, cardiac diseases, and other risk factors (y-axis). Adjusted odds ratios and 95% confidence intervals are shown for each factor (x-axis).

Several factors generally known to precipitate delirium failed to reach significance in the simple regression model, e.g., pneumonia and electrolyte disturbances (see Table 1). In the multiple regression model, the sociodemographic, functional, and neuropsychiatric factors were more relevant to the development of delirium than cardiac factors (Figure 2, Table 3). Of the sociodemographic factors and factors referring to functional impairment, institutionalization prior to admission, reduced mobility, and visual impairment increased the odds for delirium by a factor of 1.62 (95% CI, 1.00–2.65; *P* = 0.051), 3.15 (95% CI, 1.38–7.19; *P* = 0.006), and 3.10 (95% CI, 2.04–4.69; *P* < 0.001), respectively. In addition, with regards to demographic factors, high age (≥80 years) (OR, 1.81; 95% CI, 1.18–2.77; *P* = 0.006) as well as male sex (OR, 1.58; 95% CI, 1.04–2.38; *P* = 0.030) were associated with higher odds of delirium. Remarkably, the most significant risk factors were dementia and history of stroke, increasing the odds by 18.11-fold (95% CI, 5.77–56.83; *P* < 0.001) and 6.61-fold (95% CI, 1.35–32.44; *P* = 0.020). The diagnosis of substance use disorders was associated with a 3.86-fold increased risk (95% CI, 1.73–8.59; *P* = 0.001). Of the cardiac diseases, only valvular heart disease contributed to the risk for delirium (OR, 1.57; 95% CI, 1.01–2.44; *P* = 0.044). Particularly, catheter interventions were not associated with increased rates of delirium (OR, 1.39; 95% CI, 0.91–2.12; *P* = 0.124). Pressure ulcers were significantly associated, with 3.62-fold increased odds of delirium (95% CI, 1.06–12.35; *P* = 0.040). The detailed results of the multivariate analysis are shown in Table 3.

**Table 3:** Multiple regression model for the predisposing and precipitating factors for delirium in cardiology.

	**B (SE)**	**OR**	**95% CI lower-upper**	***P***
Institutionalized prior to admission	0.48 (0.22)	1.62	1.00–2.65	0.051
Emergency admission	0.05 (0.22)	1.05	0.68–1.62	0.811
Male sex	0.45 (0.21)	1.58	1.04–2.38	0.03
Age 80 years or more	0.59 (0.22)	1.81	1.18–2.77	0.006
***Functional impairment***				
Reduced mobility	1.15 (0.42)	3.15	0.38–7.19	0.006
Visual impairment	1.13 (0.21)	3.1	2.04–4.69	<0.001
Hearing impairment	0.07 (0.22)	1.08	0.69–1.67	0.746
***Psychiatric disorders***				
Dementias/degenerative disorders	2.90 (0.58)	18.11	5.77–56.83	<0.001
Substance use disorder	1.35 (0.41)	3.86	1.73–8.59	0.001
***Cardiac diseases***				
Cardiac arrest	−0.07 (0.59)	0.94	0.29–2.97	0.911
Heart failure	0.36 (0.20)	1.43	0.96–2.14	0.078
Cardiomyopathy	−0.45 (0.39)	0.64	0.29–1.37	0.249
Ischemic heart disease	−0.03 (0.22)	0.97	0.62–1.50	0.883
Valvular heart disease	0.45 (0.22)	1.57	1.01–2.44	0.044
***Other risk factors***				
Atherosclerosis	0.19 (0.29)	1.21	0.69–2.12	0.515
History of stroke	1.89 (0.81)	6.61	1.35–32.44	0.02
Pleural effusions	0.69 (0.55)	2	0.68–5.94	0.21
Pressure ulcers	1.29 (0.63)	3.62	1.06–12.35	0.04
Cardiac intervention	0.33 (0.21)	1.39	0.91–2.12	0.124
Intensive care management	0.61 (0.28)	1.84	1.04–3.23	0.035
Sepsis/SIRS	0.13 (0.45)	1.14	0.47–2.79	0.767
Liver failure	0.84 (0.81)	2.31	0.47–11.28	0.299
Cystitis	0.49 (0.27)	1.64	0.97–2.77	0.065

*Cox-Snell and Nagelkerke r^2^ = 0.19 and 0.31, SE, standard error; OR, odds ratio; CI, confidence interval*.

### Prediction Model

**Figure 3:**
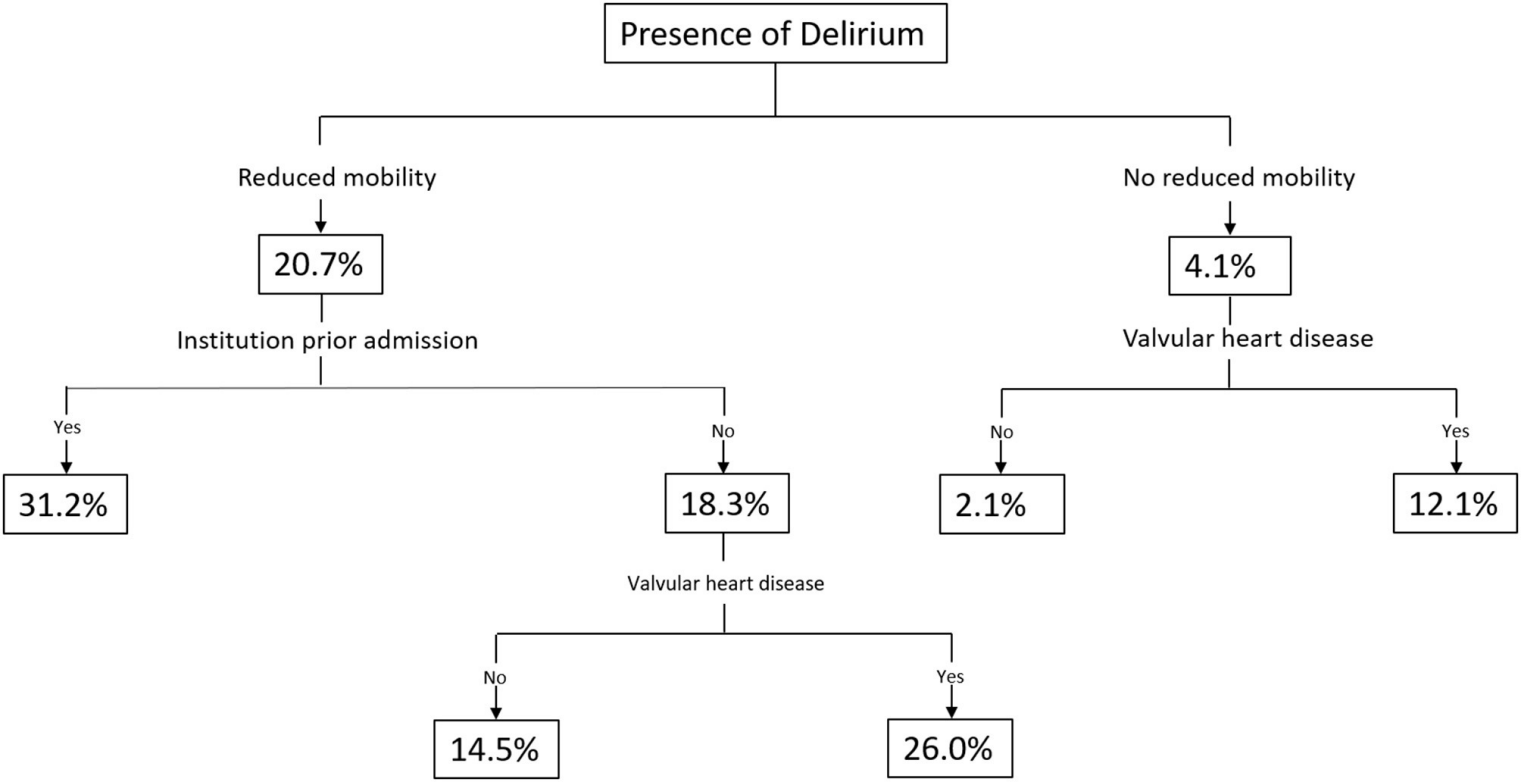
Prediction tree for the presence of delirium.

In the decision tree analysis, the predicted probability (PP) to develop delirium was revealed to be highest in patients with reduced mobility who were admitted from institutionalized living (PP = 31.2%, *P* = 0.001). The probability to develop delirium was lowest in patients with normal mobility and without valvular heart disease (PP, 2.1%; *P* = 0.001) (Figure 3).

## Discussion

### Summary of Main Findings

This study systematically assessed the association between the presence of delirium at admission or occurring within the first 3 days of hospitalization and its predisposing and precipitating factors in a large cohort of patients admitted to a cardiology ward in a tertiary care center. The prevalence of delirium was 16.0%, and the most relevant predisposing risk factors were dementia and functional impairment, followed by history of stroke and substance use disorder, whereas pressure ulcers were identified as a significant precipitating factor.

### Geriatric Syndrome as a Risk Factor for Delirium

Our findings confirmed previous notions of dementia and low function as predisposing risk factors for delirium (25). In addition, disabilities associated with advanced age, i.e., reduced mobility and visual impairment, contributed to the odds of occurrence of delirium. This constellation was previously termed geriatric syndrome as compared to healthy aging, which is characterized by an active lifestyle and physical activity (26, 27). Therefore, delirium may not be viewed as an unavoidable complication in the elderly with cardiac disease but appreciated as a potential complication when an acute illness meets unhealthy aging.

### Stroke and Delirium

Acute ischemic and hemorrhagic stroke can cause delirium, termed post-stroke delirium (28). In this study, a history of stroke was identified as a major risk factor in cardiac patients, with a 6.61-fold increased risk for developing delirium, which is a novel finding in patients admitted to cardiology wards. Recently, a systematic review has shown that a history of cerebrovascular events was associated with an increased risk of postoperative delirium (29). This association is likely related to the high rates of even mild cognitive impairment occurring after stroke (30), which can exacerbate and lead to delirium in the presence of other risk factors and hospitalization for cardiological disease.

### Medical Factors Associated With Delirium

Pressure ulcers emerged as a precipitator for delirium, a finding which is novel for patients admitted to cardiology wards. This finding is likely associated with a higher presence of pressure ulcers in patients with a geriatric syndrome (31). However, given the significance in the multivariate analysis, this association could possibly be explained by systemic inflammation and pain present in pressure ulcers, even when the criteria for sepsis and SIRS are not met (32). Pressure ulcers are very common but generally under recognized in patients admitted to internal medicine departments (33). The association with delirium that we found here supports the need for skin examination in patients at risk for pressure ulcers. In patients admitted to this cardiology ward, substance use disorders (OR, 3.86) did not reach the factor of 5.7 for alcohol use disorders previously described in elderly cohorts (3). However, the substances were not further broken down to individual classes or analyzed as to whether their chronic use or withdrawal conferred a different risk. The common medical etiologies of delirium—pneumonia, cystitis, and electrolyte imbalances—failed to reach significance in cardiology patients. One reason might be that the patients admitted to the cardiology ward of this tertiary care hospital are primarily hospitalized for the management of cardiac diseases and have less often acute infectious diseases. Regarding the sex distribution of delirious patients, i.e., male patients developed delirium more often than females, our findings resemble those of a study on inpatients of a similar age (80 vs. 76 years; male 53.9 vs. 60.8%) (34).

### Cardiac Disease and Interventions as a Risk Factor for Delirium

Interestingly, cardiac interventions, i.e., catheter interventions, were not relevant precipitants in the multivariate analysis. The overall prevalence of delirium, with or without intervention, in our cohort of patients was higher than in a large retrospective analysis of elderly patients of a similar age (mean 81 years) undergoing transcatheter aortic valve replacement (16.0 vs. 8.0%) (35), which is likely related to the routine screening algorithm employed in our study. Regarding the ongoing debate of delirium rates in interventional therapies, our findings support a favorable outcome of interventions (12, 36). The association with valvular heart disease and delirium is novel and might be explained by older age, which predisposes a person to valve diseases. Moreover, we attribute this finding to reduced cerebral blood perfusion secondary to valve diseases (37), which is linked to delirium (38). Previous studies report a positive association of delirium with heart failure (39, 40). In our study, heart failure was close to, but did not reach, statistical significance. One explanation may be that the number of potential risk factors assessed in our study was larger and that heart failure became less relevant in the analysis when including several detrimental predisposing factors.

### Study Limitations

This study systematically assessed the impact of 27 predisposing and precipitating factors for delirium. A major drawback of the study was the missing determination of baseline cognition. Patients with a cognitive disorder yet undiagnosed could further increase the correlation of dementia and delirium incidence. Of note is the fact that cardiac arrest was used as an umbrella term based on ICD-10, and no information on duration of unconsciousness and time for resuscitation was available. The impact of cardiac surgery was not investigated in this study. Furthermore, interventions were not further evaluated with regards to the type of procedure and the technical approach. Previous studies demonstrated associations with specific procedures, e.g., transapical approach for transcatheter aortic valve implantation (41). Given that routine screening for delirium was done at admission and in the first 3 days of hospitalization, statements of delirium occurrence in the remaining time of hospitalization cannot be made.

## Conclusions

The novelty of this study was the systematic assessment of predisposing and precipitating factors for delirium in a large sample of patients admitted to the cardiology ward. This study showed that, among elderly patients admitted to a cardiology ward, reduced mobility and cognitive and functional impairment significantly increase the risk of developing delirium. Pressure ulcers and history of stroke were identified as relevant risk factors previously not described in this population. Conversely, cardiac interventions did not contribute to the development of delirium. Although delirium is a neuropsychiatric syndrome, cardiologists should familiarize with it so as to apply preventive strategies, allow the early detection, and initiate appropriate management.

## Data Availability Statement

The original contributions presented in the study are included in the article/Supplementary Material, further inquiries can be directed to the corresponding author.

## Ethics Statement

The studies involving human participants were reviewed and approved by Ethics Committee of the Canton of Zurich (No: KEK-ZH-Nr. ZH-2012-0263) and conforms to the ethical guidelines of the 1975 Declaration of Helsinki. Written informed consent for participation was not required for this study in accordance with the national legislation and the institutional requirements.

## Author Contributions

The concept of this study was conceived by CZ, FH, and SB. Data were acquired by CZ, FH, BH, SB, JE, HP, MS, and KJ. The critical appraisal of the method was conceived by CZ, FH, SB, JD, RK, and MS. CZ prepared the first draft of the manuscript. All authors provided edits and critiqued the manuscript for intellectual content.

## Conflict of Interest

The authors declare that the research was conducted in the absence of any commercial or financial relationships that could be construed as a potential conflict of interest.

## Publisher's Note

All claims expressed in this article are solely those of the authors and do not necessarily represent those of their affiliated organizations, or those of the publisher, the editors and the reviewers. Any product that may be evaluated in this article, or claim that may be made by its manufacturer, is not guaranteed or endorsed by the publisher.
