# Supplementary material for: Predisposing and Precipitating Risk Factors for Delirium in Elderly Patients Admitted to a Cardiology Ward: An Observational Cohort Study in 1,042 Patients
Source: Front Cardiovasc Med. 2021 Sep 29;8:686665. doi: 10.3389/fcvm.2021.686665 (PMC8513394; doi:10.3389/fcvm.2021.686665)
Supplement: Supplementary file 1 [file Table_1.docx]

Supplementary Table 1. ICD-10 diagnostic clusters with the respective ICD-10 diagnoses included

| Sepsis / SIRS | A40-A41, R65 |
| --- | --- |
| Dementias / degenerative cerebral disorders | F00 Alzheimer’s disease  F01 vascular dementias  F02 Dementia due to elsewhere defined disorders  F03 Dementia not elsewhere defined  G30 Alzheimer’s disease  G31-.0 localized atrophies (frontal temporal dementia)  G31-.1-2 senile and alcohol-induced degenerations  G31.8-9 Degenerations ned  G32 degenerations due to elsewhere defined disorders |
| Electrolyte disturbances | E87 |
| Substance use disorders | F10-19 |
| Ischemic insults | G46 cerebral vascular syndromes  I63 cerebral insults / strokes |
| Valvular heart disease | I08 |
| Ischemic heart disease | I20-25 |
| Cardiomyopathy | I42-43 |
| Cardiac arrest | I46 |
| Cardiac insufficiency | I50 |
| Atherosclerosis | I70 |
| Pneumonia | J09-J18 |
| Respiratory disease | J30-J39, J40-J47, J95-J99 |
| Pleural effusions | J90 |
| Liver failure | K70-K72 |
| Pressure ulcers | L89 |
| Kidney disease | N00-N19, N25-N29 |
| Cystitis | N30 |
|  |  |
